# Circulating MicroRNAs as Biomarkers in Hepatocellular Carcinoma Screening: A Validation Set From China

**DOI:** 10.1097/MD.0000000000000603

**Published:** 2015-03-13

**Authors:** Li Jiang, Qi Cheng, Bin-Hao Zhang, Ming-Zhi Zhang

**Affiliations:** From the Department of Biliary and Pancreatic Surgery (LJ); Hepatic Surgery Center, Affiliated Tongji Hospital, Tongji Medical College, Huazhong University of Science and Technology, Wuhan, China (QC,B-HZ); and Departments of Medicine and Cancer Biology, Vanderbilt University School of Medicine, Nashville, Tennessee (M-ZZ).

## Abstract

Hepatocellular carcinoma (HCC) is a global public health concern. Current diagnostic methods show poor performance in early-stage HCC detection. Accumulating evidences revealed the great potential of microRNAs (miRNAs) as noninvasive biomarkers in HCC detection. In this study, we examined the diagnostic performance of serum miR-10b, miR-106b, and miR-181a for HCC screening in China. Furthermore, a systematic review of previous related studies was conducted to confirm our results.

One hundred eight participants including 27 HCC patients, 31 chronic liver disease (CLD) patients, and 50 healthy people were recruited in this study. Blood specimen was drawn from each participant to extract serum miRNAs. Statistical analyses were performed to assess the 3 miRNAs levels in HCC, CLD patients, and normal controls. A meta-analysis was conducted to further assess the diagnostic value of miRNAs in HCC detection based on previous studies.

All these miRNAs (miR-10b, miR-181a, miR-106b) could well discriminate HCC patients from normal controls, with area under the receiver-operating characteristic curve (AUC) values of 0.85 (95% confidence interval [CI]: 0.76–0.94), 0.82 (95% CI: 0.72–0.91), and 0.89 (95% CI: 0.81–0.97), respectively. In addition, these miRNAs could distinguish HCC cases from CLD controls with a medium accuracy. However, the ability of these miRNAs in differentiating CLD patients from normal controls was not satisfactory. Panel of these miRNAs displayed a better performance compared with single miRNA assay, with AUC values of 0.94 (95% CI: 0.89–0.99) in discriminating HCC patients from normal controls and 0.91 (95% CI: 0.80–0.97) in discriminating HCC patients from CLD controls. Results of meta-analysis of previous studies combined with the current study suggested that circulating miRNAs could well differentiate HCC from normal controls, with AUC values of 0.86 (95% CI: 0.82–0.89) for single miRNA assay and 0.94 (95% CI: 0.91–0.96) for miRNA panel assay.

Serum miR-10b, miR-106b, and miR-181a have great potential to serve as accurate and noninvasive biomarkers for HCC preliminary screening. Meta-analysis of previous studies combined with current study further confirmed that circulating miRNAs could play an important role in HCC detection. Further large-scale studies are needed to confirm the clinical significance of circulating miRNAs in HCC screening.

## INTRODUCTION

Hepatocellular carcinoma (HCC) is one of most prevalent malignancies worldwide with high mortality. According to latest GLOBOCAN 2012 report, there are approximately 782,000 new cases and 746,000 deaths in 2012.^1^ Most of the HCC cases occur in less-developed regions, especially in China. China accounts for over half of HCC cases and deaths in the world.^2^ The carcinogenesis of HCC is reported to be associated with chronic liver diseases, infection with hepatitis B or C virus, as well as excessive consumption of alcohol.^3^ Nonetheless, the underlying mechanism is not still well elucidated. The overall 5-year survival rate for HCC patients remains very low, ranging from 5% to 9%.^4,5^ The high mortality rate of HCC is mainly due to late diagnosis and lack of effective treatments. However, the 5-year survival rate will increase to 69%, if HCC patients are diagnosed at early stage.^6,7^ Therefore, the most urgent need is to discover accurate diagnostic techniques for early-stage HCC.

Medical imaging technologies such as ultrasound, computed tomography (CT) scan, magnetic resonance imaging (MRI), etc, have been widely utilized in HCC detection.^8^ Advances in medical imaging technology have contributed to better characterization of hepatic lesions in HCC patients. Regardless, small tumors remain difficult to detect, particularly in the presence of cirrhosis. Ultrasound as a diagnostic technique for HCC has a sensitivity of 65% to 85% and a specificity of >90%.^9^ However, ultrasound is operator-dependent diagnostic procedure that the accuracy of the results depends on the ability of technologist to properly operate the equipment.^10^ The main disadvantage of CT scan is that it provokes a risk of radiation to patients. MRI is a highly sensitive imaging technique for HCC detection, whereas the high cost of equipment may limit its utilization in cancer diagnosis.^11^ Percutaneous biopsy can provide definitive evidences of disease when the imaging results are uncertain.^12^ But it may cause discomfort or pain during the procedure. Alpha fetoprotein (AFP) level can serve as a useful tumor marker for HCC diagnosis. The false-negative rate of AFP screening test may be as high as 40% for early-stage HCC detection.^13^ Even in patients with advanced-stage HCC, AFP screening test still has 15% to 30% false-negative rate.^14^ Thus, noninvasive and accurate biomarkers are urgently needed for HCC diagnosis. Previous studies have examined the diagnostic performance of miRNAs as novel biomarkers for HCC detection.

MicroRNAs (miRNAs) are a class of small, noncoding, approximately 22-nucleotides-long RNAs, which may function as post-transcriptional regulator of gene expression. An estimated 30% of all protein-coding genes are regulated by miRNAs.^15^ MiRNAs bind to the 3’-untranslated region of messenger RNAs, leading to translational repression or mRNA degradation. Several studies indicate that miRNAs are involved in a variety of physiological processes, including cell proliferation, differentiation, metabolism, and apoptosis etc. It has been reported that the change in miRNAs expression may correlate with pathogenesis of cancer.^16^ In addition, miRNAs are detectable and remarkably stable in clinical samples like blood, serum, plasma, urine, and feces. Furthermore, miRNAs are shown to be resistant to endogenous RNase activity, extreme pH, high temperature, and multiple freeze-thaw cycles. These findings may suggest that miRNAs can serve as a promising biomarker in cancer detection.

In previous studies, it has been reported that overexpression of miR-10b is closely related to the metastasis and invasion of breast cancer cells.^17^ MiR-10b is also implicated in the development of glioblastoma, gastric cancer, and pancreatic cancer.^18–20^ MiR-106b is identified as an oncogene in various cancers such as gastric cancer, bladder cancer, and laryngeal carcinoma.^21–23^ MiR-181a promotes gastric cancer cell proliferation and inhibits apoptosis probably by repressing the expression of tumor suppressor KLF6.^24^ Moreover, miR-181a modulates TGF-β signaling pathway to induce epithelial-to-mesenchymal transition (EMT), which plays important role in cancer metastasis.^25^ A number of studies have also reported abnormal expression of miRNAs as well in chronic hepatitis patients. However, few studies have been performed to investigate the expression level of miR-10b, -106b, and -181a in chronic hepatitis patients. Moreover, although some studies have examined the association between the aberrant expression of miRNAs and the progression of HCC, there were still several inconsistent conclusions.

In efforts to assess the diagnostic performance of miRNAs in HCC screening, we first selected 3 candidate miRNAs (miR-10b, miR-106b, and miR-181a) to quantify their expressions in HCC patients compared with CLD patients and healthy volunteers and investigate whether these 3 miRNAs in serum could screen HCC patients. In addition, we conducted a meta-analysis of previous related studies combined with the current study, aiming to evaluate comprehensively the diagnostic value of miRNAs for HCC detection.

## MATERIALS AND METHODS

### Study Population

A total of 108 participants, which consist of 27 HCC patients, 31 CLD patients and 50 healthy volunteers, were recruited from the Affiliated Tongji Hospital, Tongji Medical College between January 2014 and December 2014. Enrolled participants have to meet the criteria as follows: diagnosis of HCC confirmed by histopathological examination or biopsy; patients without previous history of cancer; patients without previous history of receiving chemotherapy or radiotherapy. Tumor-node-metastasis (TNM) staging system was used to determine the stage of tumors (TNM I, II, III, or IV) on the basis of their status of metastasis (yes or no), distant metastasis (yes or no), tumor size (≤5 cm or >5 cm). Clinicopathological features of patients including age, sex, hypertension, diabetes mellitus, alcohol consumption, as well as tobacco smoking were provided in Table 1. The study was approved by the Ethics Committee of the Affiliated Tongji Hospital, Tongji Medical College, in compliance with the Declaration of Helsinki and regulatory laws in China. Written informed consents were obtained from patients before proceeding any medical examination.

**TABLE 1:**
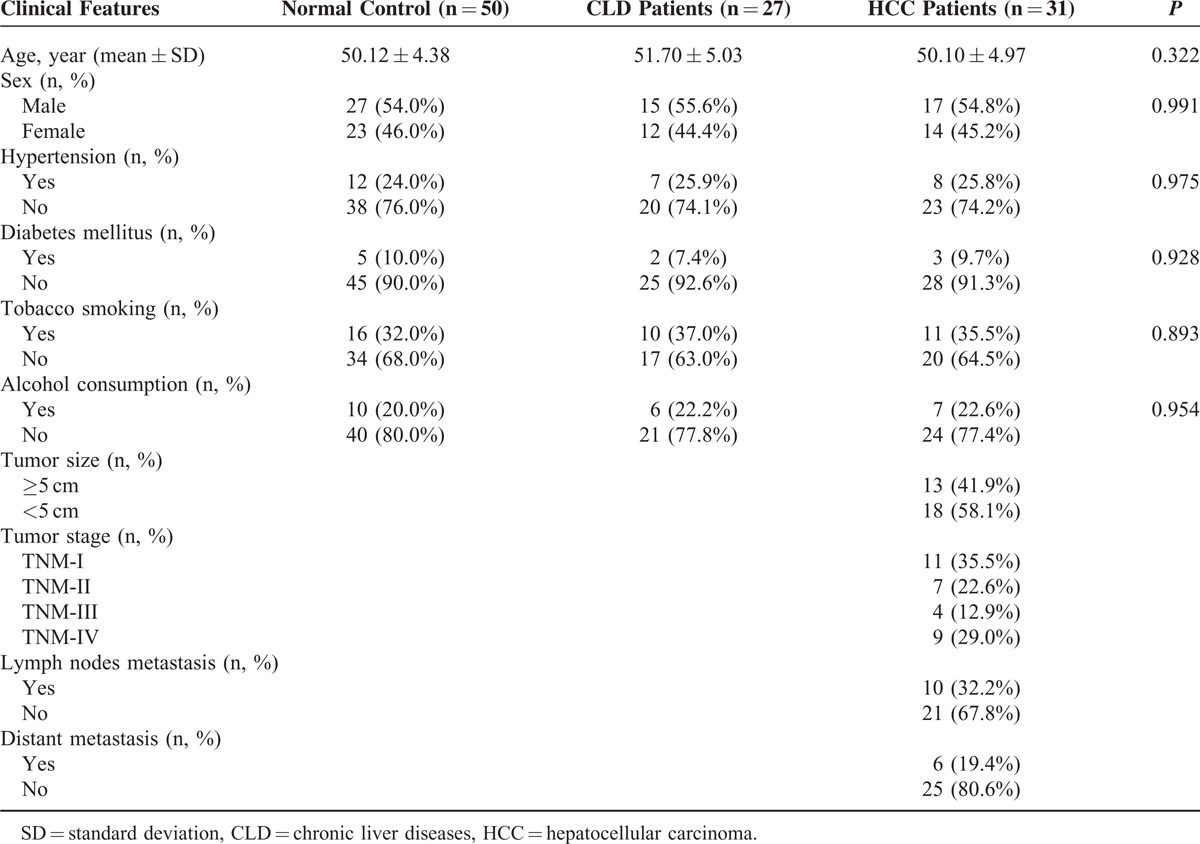
Clinical Characteristic of Subjects

### Sample Collection and RNA Extraction

Five millilitres of peripheral blood was drawn from each participant. Blood specimens were separated into supernatant and cellular sediments by centrifugation at 3000 rpm for 10 minutes. The supernatants were transferred to new centrifuge tubes and further centrifugated at 12000 rpm for 2 minutes to obtain the serum. Serum samples were then stored at −80°C before further processing. Total RNA from 400-μL blood serum was isolated using mirVana PARIS Kit (Ambion, Austin, TX, USA) in accordance with the manufacturer's protocol. RNA was eluted by 10 μL of RNase-free water (Ambion). The quality of RNA was assessed by measuring the absorbance at 260 and 280 nm with Nanodrop 1000A spectrophotometer (NanoDrop Technologies, Wilmington, DE, USA).

### Quantitative Reverse-transcriptase Polymerase Chain Reaction

The expression of miRNAs was measured by quantitative reverse-transcriptase polymerase chain reaction (qRT-PCR) with human TaqMan MicroRNA Assay Kits (Applied Biosystems, Foster City, CA, USA). Reverse transcription reaction was performed using TaqMan MicroRNA Reverse Transcription Kit (Applied Biosystems). Reaction mixture for cDNA synthesis was prepared, which was comprised of 5-μL RNA extract, 0.15 μL of 100-mmol/L dNTPs, 3 μL of reverse transcription primers, 1.5 μL of 10X reverse transcription buffer, 0.19 μL of 20-U/μL RNase Inhibitor, 1 μL of 50-U/μL Multiscribe Reverse Transcriptase, and 4.16 μL of nuclease-free water. Reaction solutions for cDNA synthesis were incubated at 16°C for 30 minutes, subsequently at 42°C for 30 minutes, and then at 85°C for 30 minutes, and ultimately held at 4°C. qRT-PCR was conducted in a total volume of 20 μL, which consisted of 1.33-μL cDNA after reverse transcription reaction, 10-μL TaqMan Universal PCR Master Mix II (2X) without UNG reagent (Applied Biosystems), 1-μL specific primers and 7.67-μL nuclease-free water per reaction. Expression of different miRNAs was measured using Bio-Rad IQ5 s system (Bio-Rad Laboratories, Inc). In the first step, cDNA was denatured by heating to 95°C. MicroRNAs were amplified for 45 cycles at 95°C for 15 s and at 60°C for 60 s. Cycle threshold (Ct) values were calculated by Bio-Rad IQ5 2.1 Standard Edition Optical System Software 2.1.94.0617. U6 snRNA as the internal reference was used to compare the expression level of miRNAs. Relative expression level of miRNAs can be defined as 2^−ΔΔCt^, where ΔCt was the difference of Ct values between miRNAs and U6 snRNA.

### Statistical Analysis

All statistical analyses were carried out with Stata 12.0 software. MiRNAs expressions among different groups (HCC patients, CLD patients, and healthy volunteers) were assessed by 1-way analysis of variance (ANOVA) test. Clinical features (age, sex, hypertension, diabetes mellitus, tobacco smoking, and alcohol consumption) were also analyzed by 1-way ANOVA test or chi-square (*χ*^2^) test. A *P* value <0.05 was considered statistically significant. Receiver-operating characteristics (ROC) curves were established by plotting sensitivity against 1-specificity. The diagnostic values of 3 candidate miRNAs were evaluated by area under the ROC curve (AUC). Multivariable logistic regression was used to combine these miRNAs and further calculate the diagnostic value of the miRNA panel. Graphs were generated with GraphPad Prism 5.0 (GraphPad Software Inc, San Diego, CA, USA).

For the meta-analysis, we conducted a comprehensive literature search in PubMed, Embase, the Cochrane Library, and other sources before November 20, 2014. The pooled diagnostic parameters, including sensitivity, specificity, positive likelihood ratio (PLR), negative likelihood ratio (NLR), and diagnostic odds ratio (DOR), were calculated using the bivariate meta-analysis model. A pooled summary receiver-operating characteristics (SROC) curve was plotted, and the AUC was calculated.

## RESULTS

### Characteristics of Study Population

As shown in Table 1, 108 subjects (31 HCC patients, 27 CLD patients, and 50 healthy controls) were recruited in the current study. No significant difference was observed in age, sex, and clinical features among 3 groups (all *P* > 0.05). HCC patients were categorized based on the tumor size, 13 with tumor ≥5 cm, and 18 with tumors <5 cm. According to TNM Stages, 11 were diagnosed as having stage I HCC, 7 were identified as stage II HCC, 4 were determined as stage III, and 9 were at stage IV. In the light of metastasis status, there are 10 patients with tumor metastasis, 21 patients without tumor metastasis. Among 10 patients with tumor metastasis, 6 of them were identified as distant metastasis.

### Expression Patterns and Diagnostic Accuracy of MiR-10b

**FIGURE 1:**
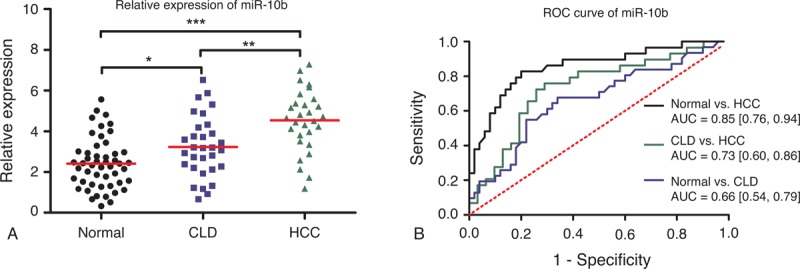
Diagnostic performance of miR-10b. **(**A) Relative expression levels of miR-10b in normal controls, CLD, and HCC patients. Relative expression levels of miR-10b have standardized. ^∗^*P* < 0.05; ^∗∗^*P* < 0.01; ^∗∗∗^*P* < 0.001. (B) Receiver-operating characteristic (ROC) curve of miR-10b in 3 groups (HCC vs normal; CLD vs normal; HCC vs CLD). AUC values are presented by the estimate with 95% confidence interval. AUC = the area under the summary ROC curve, CLD = chronic liver diseases, HCC = hepatocellular carcinoma, ns = nonsignificant.

The expression level of miR-10b was examined in serum samples collected from 108 subjects. The scatter dot plot in Figure 1A illustrated the relative expression of miR-10b in HCC patients, CLD patients, and healthy controls. HCC patients have a significantly higher expression than normal control (*P* < 0.001) and CLD patients (*P* < 0.01). In addition, miR-10b is also moderately upregulated in CLD patients compared with healthy controls (*P* < 0.05). The diagnostic accuracy of miR-10b was measured by ROC curves in Figure 1B and the corresponding AUC values were 0.85 (95% CI: 0.76–0.94) for differentiating HCC patients from normal individuals, 0.73 (95% CI: 0.60–0.86) for differentiating HCC from CLD patients, and 0.66 (95% CI: 0.54–0.79) for differentiating CLD patients from normal individuals.

### Expression Patterns and Diagnostic Accuracy of MiR-181a

**FIGURE 2:**
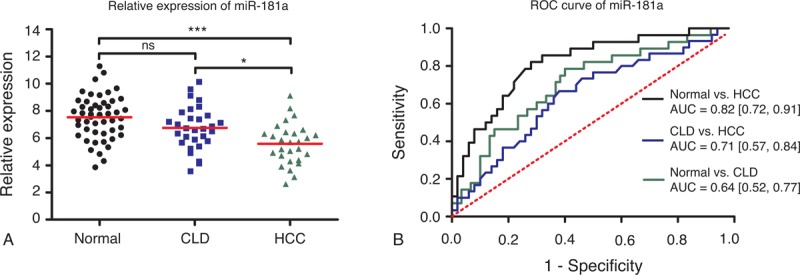
Diagnostic performance of miR-181a. **(**A) Relative expression levels of miR-181a in normal controls, CLD, and HCC patients. Relative expression levels of miR-10b have standardized. ^∗^*P* < 0.05; ^∗∗^*P* < 0.01; ^∗∗∗^*P* < 0.001. (B) Receiver-operating characteristic (ROC) curve of miR-181a in 3 groups (HCC vs normal; CLD vs normal; HCC vs CLD). AUC values are presented by the estimate with 95% confidence interval. AUC = the area under the summary ROC curve, CLD = chronic liver diseases, HCC = hepatocellular carcinoma, ns = nonsignificant.

In Figure 2A, it is revealed that the expression levels of miR-181a in HCC patients were significantly lower than CLD patients (*P* < 0.05) and normal controls (*P* < 0.001). No significant difference in miR-181a expression was found between CLD patients and normal controls. As shown in Figure 2B, the diagnostic accuracy of miR-181a in HCC against normal controls (AUC = 0.82, 95% CI: 0.72–0.91) was much higher than in HCC against CLD patients (AUC = 0.71, 95% CI: 0.57–0.84). The AUC value for differentiating CLD patients from normal controls was even lower with an AUC of 0.64 (95% CI: 0.52–0.77).

### Expression Patterns and Diagnostic Accuracy of MiR-106b

**FIGURE 3:**
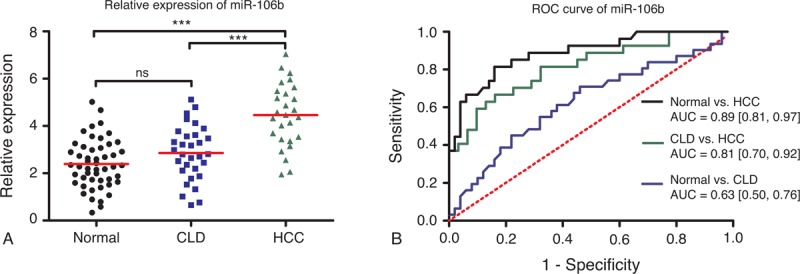
Diagnostic performance of miR-106b. **(**A) Relative expression levels of miR-106b in normal controls, CLD, and HCC patients. Relative expression levels of miR-10b have standardized. ^∗^*P* < 0.05; ^∗∗^*P* < 0.01; ^∗∗∗^*P* < 0.001. (B) Receiver-operating characteristic (ROC) curve of miR-106b in 3 groups (HCC vs normal; CLD vs normal; HCC vs CLD). AUC values are presented by the estimate with 95% confidence interval. AUC = the area under the summary ROC curve, CLD = chronic liver diseases, HCC = hepatocellular carcinoma, ns = nonsignificant.

As shown in Figure 3A, miR-106b was over-expressed in HCC patients compared with healthy volunteers (*P* < 0.001) and CLD patients (*P* < 0.001). Nonetheless, there is no evident change in miR-106b level between CLD group and normal group. Figure 3B showed that the diagnostic performance of miR-106b was relatively good in HCC against normal controls (AUC = 0.89, 95% CI: 0.81–0.97). The accuracy of miR-106b in differentiating HCC against CLD was also satisfactory (AUC = 0.81, 95% CI: 0.70–0.92), but its performance was much worse in CLD against normal controls (AUC = 0.63, 95% CI: 0.50–0.76).

### Diagnostic Accuracy of the Combination of MiR-10b, MiR-106b, and MiR-181a

**FIGURE 4:**
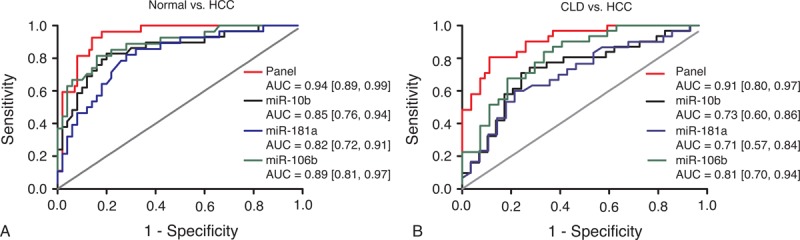
Diagnostic performance of miRNA panel comprised by miR-10b, miR-181a, and miR-106b. (A) Receiver-operating characteristic (ROC) curve of miRNA panel in differentiating HCC from normal controls. (B) ROC curve of miRNA panel in differentiating HCC from CLD controls. AUC values are presented by the estimate with 95% confidence interval. AUC = the area under the summary ROC curve, CLD = chronic liver diseases, HCC = hepatocellular carcinoma, ns = nonsignificant.

An analysis of ROC curves for this 3-serum miRNAs was performed to evaluate the diagnostic value in differentiating HCC from healthy controls and CLD patients. As shown in Figure 4A, the combination of miR-10b, miR-106b, and miR-181a had an AUC of 0.94 (95% CI: 0.89–0.99) in distinguishing the HCC group from the healthy controls. The AUC value of the miRNAs panel in differentiating HCC patients from CLD patients was 0.91 (95% CI: 0.80–0.97) (Figure 4B). The results revealed that the combination of miR-10b, miR-106b, and miR-181a presented higher diagnostic accuracy in HCC against normal controls than that against CLD cases.

### Meta-analysis of Circulating MiRNAs as Biomarkers for HCC Detection

**FIGURE 5:**
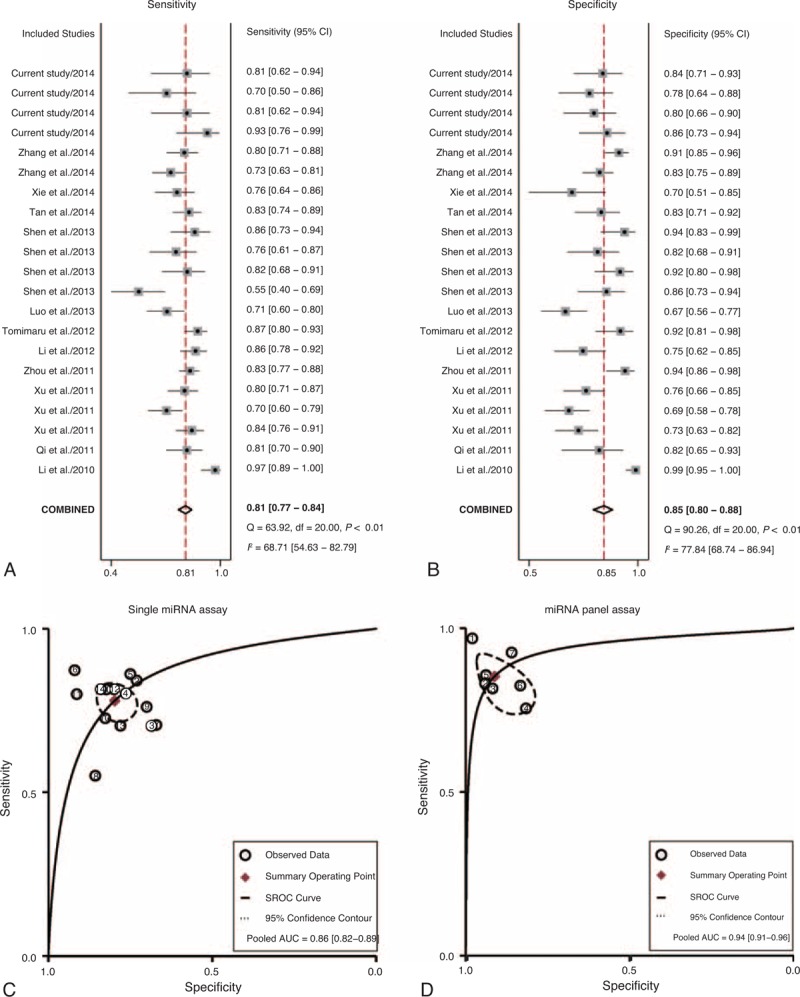
Meta-analysis of diagnostic studies in differentiating HCC from normal controls. (A) The forest plots of sensitivity in differentiating HCC from normal controls with the corresponding heterogeneity. (B) The forest plots of specificity in differentiating HCC from normal controls with the corresponding heterogeneity. The sensitivity and specificity from each study are represented by square, and the CI is indicated by error bars. (C) The SROC curves based on single miRNA assay. (D) The SROC curves based on miRNA panel assay. (○) observed data; (♦) summary operating point, (-) SROC curve, (-) 95% confidence contour. CI = confidence interval, SROC = summary receiver operator characteristic.

**FIGURE 6:**
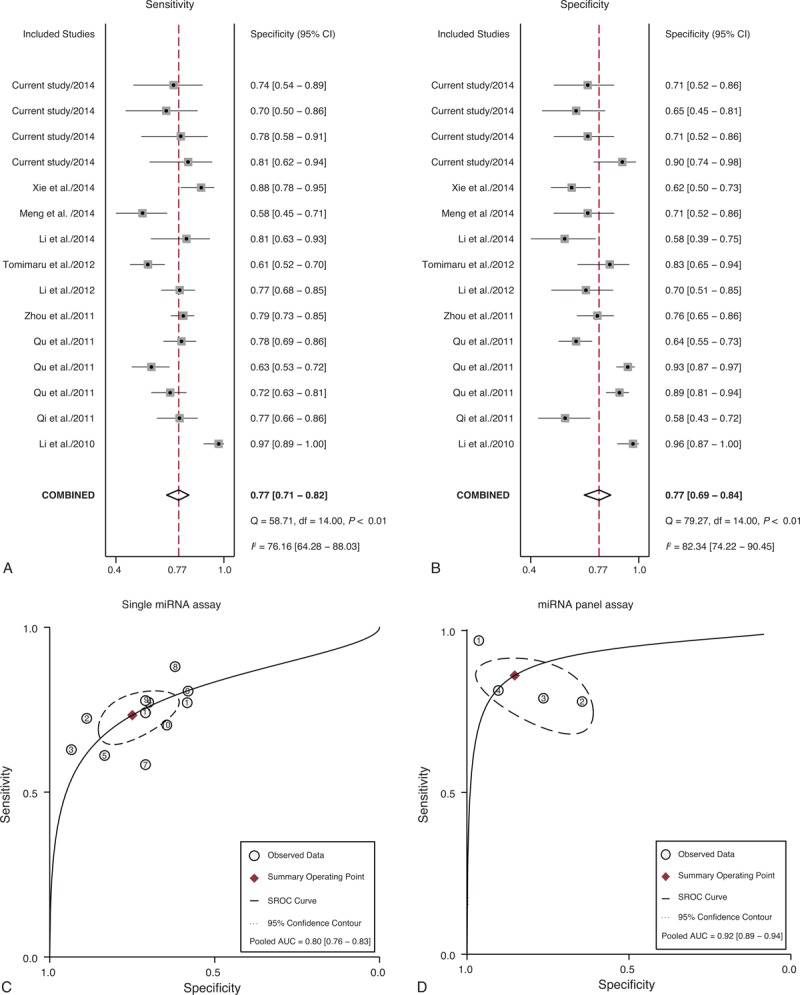
Meta-analysis of diagnostic studies in differentiating HCC from CLD controls. (A) The forest plots of sensitivity in differentiating HCC from CLD controls with the corresponding heterogeneity. (B) The forest plots of specificity in differentiating HCC from normal controls with the corresponding heterogeneity. The sensitivity and specificity from each study are represented by square, and the CI is indicated by error bars. (C) The SROC curves based on single miRNA assay. (D) The SROC curves based on miRNA panel assay. (○) observed data; (♦) summary operating point, (-) SROC curve, (-) 95% confidence contour. CI = confidence interval, CLD = chronic liver diseases, HCC = hepatocellular carcinoma, SROC = summary receiver operator characteristic.

The literature search finally yielded a total of 15 eligible studies for this meta-analysis.^26–39^ The general characteristics of the included articles are listed in Table 2.^26–39^ The forest plots illustrated the pooled sensitivity (Figure 5A) and specificity (Figure 5B) in differentiating HCC from normal controls. The meta-analysis results suggested that circulating miRNAs could well differentiate HCC from normal controls, with AUC values of 0.86 (95% CI: 0.82–0.89) for single miRNA assay (Figure 5C) and 0.94 (95% CI: 0.91–0.96) for miRNA panel assay (Figure 5D). The pooled sensitivity, specificity, PLR, NLR, and DOR were summarized in Table 3. Similarly, Figure 6A and B presented the pooled sensitivity and specificity for differentiating HCC from CLD. The SROC curve analyses also suggested a relatively high overall diagnostic accuracy, with AUC values of 0.80 (95% CI: 0.76–0.83) for single miRNA assay (Figure 6C) and 0.92 (95% CI: 0.89–0.94) for miRNA panel assay (Figure 6D), respectively.

**TABLE 2:**
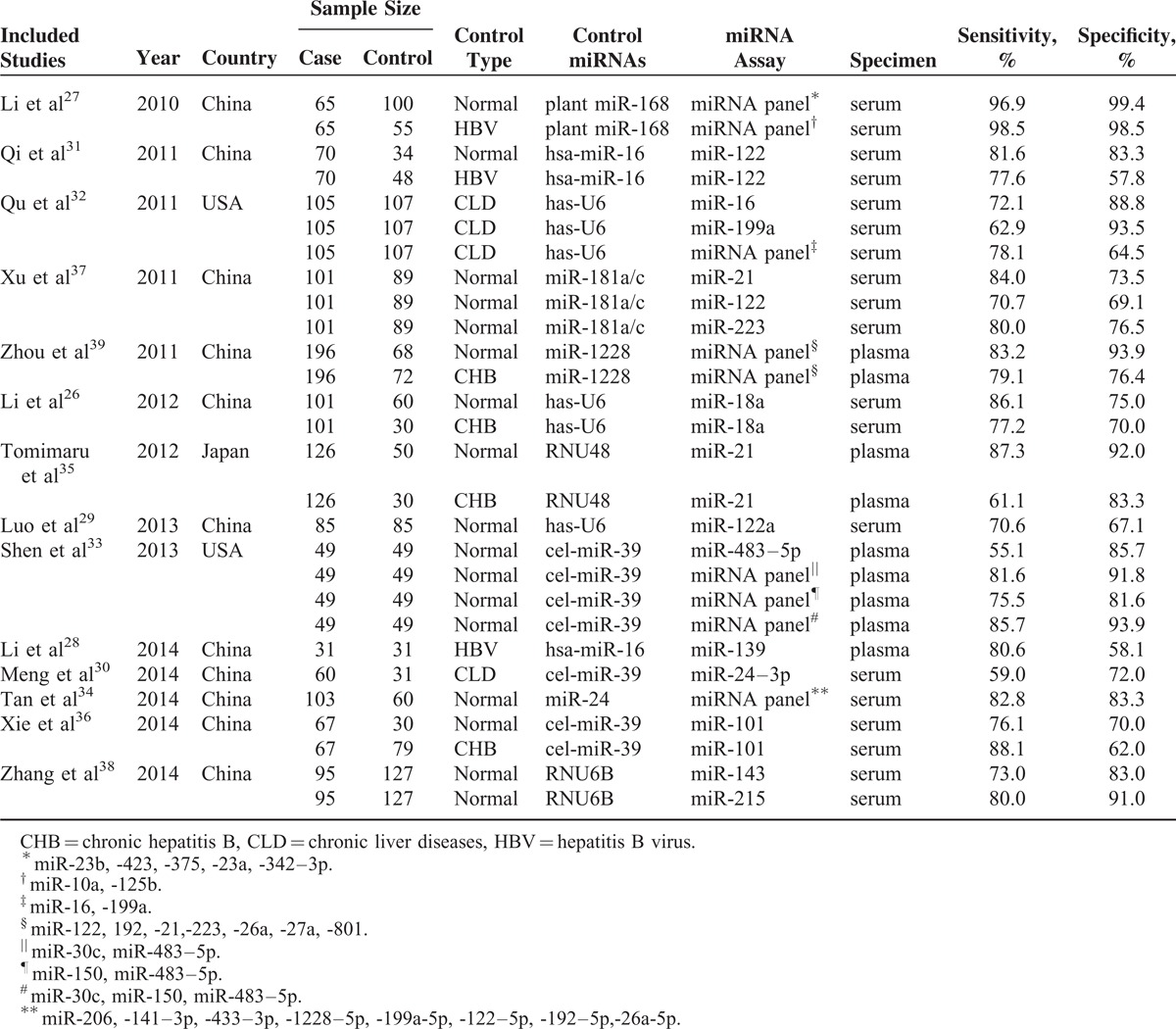
Main Characteristics of Included Studies Included in the Meta-analysis

**TABLE 3:**
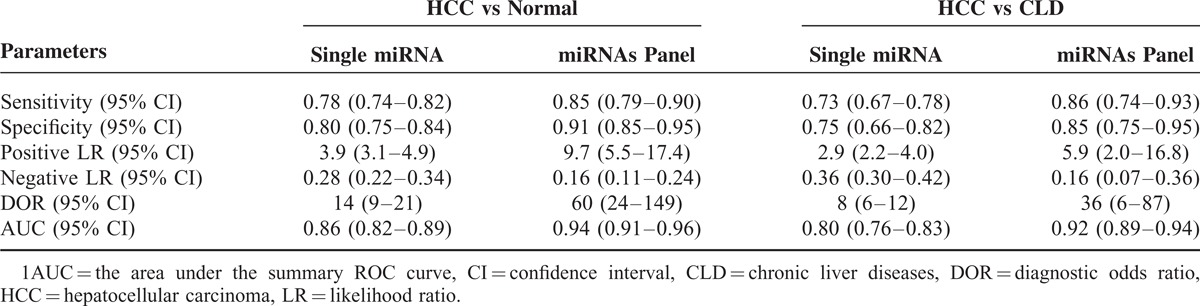
Pooled Diagnostic Accuracy of miRNAs in Discriminating HCC From Controls

## DISCUSSION

Current approaches for the HCC detection include ultrasound, CT scan, MRI, percutaneous biopsy, and AFP test. However, diagnostic performances of these techniques are not particularly satisfactory in the HCC diagnosis, thereby the late diagnosis partly contribute to the high mortality rate of HCC. The discovery of miRNA may provide a novel auxiliary screening test for HCC detection. In this study, we examine the level of 3 miRNAs (miR-10b, miR-106b, and miR-181a) in HCC patients, CLD patients, and healthy controls. Previous studies have reported a broad range of dysregulated miRNAs implicated in carcinogenesis and progression of HCC and evaluated their diagnostic performance. For instance, miR-21 regulates multiple biological processes such as cell proliferation, apoptosis, or tumor invasiveness by targeting PTEN, PDCD4, and RECK in HCC.^40^ High level of miR-143 expression can promote tumor metastasis by FNDC3B repression.^41^ MiR-101 promotes the progression of cancer via modulating Mcl-1.^42^ In the current study, our data indicated that serum miR-10b, miR-106b, and miR-181a showed remarkably high diagnostic accuracy in differentiating HCC cases from healthy controls, and their combinations have an even better performance. The miRNA panel assay exhibited a higher diagnostic performance compared with single miRNA assay. Taken together, our study suggested that miR-10b, miR-106b, and miR-181a have potential value as noninvasive biomarkers in HCC preliminary screening, especially using the combination of the 3 miRNAs.

MiR-10b has been reported to function as onco-miR in a variety of cancer such as breast cancer,^43^ colorectal cancer,^44^ esophageal cancer,^45^ and pancreatic cancer.^46^ Patients with cancer mentioned above have a elevated level of miR-10b. Our results are consistent with the previous studies, which suggest that miR-10b may have the potential to serve as a universal tumor marker in various cancers. In this study, a significant upregulation of miR-10b was also identified in patients with CLD, compared with healthy controls. Patients with HCC appeared to have a much higher level of miR-10b than patients with CLD. These findings may imply that elevated miR-10b is probably associated with inflammation, which may result from liver tissue injury.^37^ Both HCC and CLD may cause liver tissue injury to different degrees. Similarly, overexpression of miR-106b was observed in cancer patients. Furthermore, miR-106b promotes cell proliferation through regulation of p21 and E2F5 target gene. In this study, upregulation of miR-106b was also found in HCC patients. Our results revealed that miR-106b could well discriminate HCC from CLD and normal controls, with the higher diagnostic performance compared with the other 2 miRNAs. Unlike miR-10b and miR-106b, miR-181a has lower expression in HCC patients than normal controls, which highlighted its tumor-suppressive role in carcinogenesis.^47,48^

We further conducted a meta-analysis of the previous published articles and the present study to evaluate diagnostic value of miRNAs in HCC detection. In this meta-analysis, the overall results revealed that circulating miRNAs could discriminate HCC from CLD and normal controls with a relatively high accuracy, especially the miRNA panel assay. It is suggested that miRNAs may serve as promising diagnostic biomarker for differentiating HCC patients from both healthy controls and CLD cases.

Our study is the first report to investigate the great potential of miR-10b, miR-106b, miR-181a, and their combinations as biomarkers in HCC detection. Furthermore, we perform a meta-analysis to summarize the present study with the published data. However, there are still several limitations. The selection of internal reference may have great impact on the reliability of RT-PCR results, which could also lead a potential heterogeneity in the meta-analysis. Unfortunately, there is no widely accepted universal internal reference for RNA quantification. U6 snRNA was selected as internal control for RT-PCR in our new study, whereas there are several different types of internal reference used in other included studies. Notably, the sample size of participants was relatively small. Large population-based investigation should be further performed to validate the results. Besides, only 2 of the 14 previous articles were conducted in white populations, whereas the remaining articles were performed in Asian populations. Therefore, it is unclear whether ethnicity exerts influence on the diagnostic performance of miRNAs or not.

In summary, the current study indicated that the 3 single serum miRNAs (miR-10b, miR-106b, and miR-181a) and the 3-miRNAs panel are able to serve as accurate and noninvasive biomarkers for HCC preliminary screening. Furthermore, meta-analysis of previous studies combined with current study further confirmed that circulating miRNAs could play an important role in HCC detection. Further large-scale studies are needed to confirm the clinical significance of circulating miRNAs in HCC screening.
